# Understanding neurodegeneration and regeneration in the CNS: New concepts, clinical investigations, and an unsung hero of neuroscience

**DOI:** 10.1002/nep3.70058

**Published:** 2026-08-18

**Authors:** Piotr Walczak, Shen Li, Xunming Ji, Johannes Boltze

**Affiliations:** ^1^ Center for Advanced Imaging Research, Department of Diagnostic Radiology and Nuclear Medicine University of Maryland Baltimore Maryland USA; ^2^ Department of Neurology and Psychiatry, Beijing Shijitan Hospital Capital Medical University Beijing China; ^3^ Chinese Academy of Medical Sciences and Peking Union Medical College Beijing China; ^4^ Beijing Institute of Brain Disorders Capital Medical University Beijing China; ^5^ School of Life Sciences University of Warwick Coventry UK

**Keywords:** Alzheimer disease, brain injuries, brain ischemia, cell tracking, circadian rhythm, cognitive dysfunction, coronavirus, epilepsy, heparitin sulfate, ischemic, ischemic conditioning, neurodegenerative diseases, neuroglia, neuroinflammatory diseases, Parkinson disease, stroke, traumatic

Neurodegeneration and neuroregeneration are main elements of many central nervous system (CNS) conditions, and therapeutic strategies are trying to target these. Nevertheless, there is still a concerning lack of understanding of underlying mechanisms and their contribution to both degenerative and regenerative processes, making clinical translation of novel treatment concepts challenging. However, such understanding is decisive, especially since neuroregenerative approaches or interventions that foster functional recovery are often applied in the context of clinically established therapies and not as stand‐alone approaches.^1^ This constellation automatically increases potential interactions and confounders while making the detection of add‐on benefits to established treatment a considerable statistical challenge. At the same time, there is a need to improve the treatment standards of frequent neurological conditions across the globe. The current issue of *Neuroprotection* introduces contributions aiming to increase our understanding of neurodegeneration and tissue as well as functional regeneration. It also reports results from clinical investigations of CNS‐related COVID‐19 infection sequelae, such as cognitive decline. It further features a technical advancement for in vitro analyses and presents an interesting historical commentary. The spectrum of topics discussed is very wide, thus well representing the broad scope of the journal. Nevertheless, there is one element all contributions have in common: they aim to increase our understanding of important aspects of translational and clinical neuroscience from the past, at present, and hopefully in the future.

Chen and Steinberg present an editorial that discusses an interesting concept. They propose that limb laterality might have an impact on the efficacy of remote ischemic conditioning (RIC) in the context of stroke. RIC has been shown to mitigate stroke impact in several animal studies, even when applied after the ischemic event (post‐conditioning),^2^ but clinical studies so far reported inconclusive results. In clinical studies that reported unilateral RIC and indicated the treated limb, the conditioned limb was always the one not affected by the stroke. It was chosen based on practical considerations rather than an informed rationale. The authors then offer such a biological rationale for the importance of laterality. Performing RIC is a tactile stimulus as it typically involves cyclic occlusions of a cuff to generate transient peripheral limb hypoxia/ischemia. It is known that modulation of unilateral somatosensory input reduces interhemispheric inhibition of the ipsilesional motor cortex. After stroke, interhemispheric inhibition can be excessive and prevent motor recovery, so the appropriate stimulation modality might promote motor performance. Moreover, data from animal models suggest that blocking afferent neuronal signaling abolishes RIC effects after stroke. This provides a framework for an intriguing working hypothesis on the impact of laterality in post‐stroke RIC, but the reality is likely more complex, involving aspects such as time and duration of RIC or RIC intensity. Moreover, there is no detailed knowledge about other potential differences between unilateral and bilateral RIC. Based on these knowledge gaps, the authors suggest meaningful next steps for preclinical and clinical RIC research as an experimental stroke treatment. The manuscript is conceptual and hypothetic in nature, but may indeed inform interesting future studies that can lead to a better understanding of RIC as well as its therapeutic impact and limitations in the context of stroke.

Epilepsy is a major global public health issue,^3^ and its burden can reflect health care inequality in some regions. Chen and coauthors provide a comprehensive review of the topic, describing recent global trends in epilepsy incidence and treatment, adding a Chinese perspective. This is relevant since health inequality, for instance caused by urban‐rural health care gaps, leads to remarkable regional differences in epilepsy mortality. There is also a shift of epilepsy from children towards middle‐aged and aged populations, which may be caused, among other reasons, by an increasing number of stroke‐related epilepsy cases. The review further analyses epilepsy burden by considering three major determinants: biological characteristics, health care system performance, and social aspects. It also highlights challenges in specific patient populations, including children, women, elderly patients, and those suffering from rare epilepsy syndromes. By analyzing challenges in epilepsy healthcare in China and discussing already realized or potential solutions for these, the review also gives recommendations to address similar changes in other regions. Thus, the work by Chen et al. provides a robust and multi‐factorial study of high relevance for clinical practice.

Heparan sulfate is a glycosaminoglycan that is ubiquitously present in the extracellular matrix of the CNS. Unlike other glycosaminoglycans, it is known to be involved in regenerative and reparative processes after CNS injury.^4^ This led to a high interest in heparan sulfate, and current research tries to utilize heparan sulfate as well as synthetic glycomimetics to facilitate reparative processes after stroke and in other neurodegenerative disorders. Nevertheless, there are still significant knowledge gaps regarding how exactly heparan sulfate orchestrates reparative processes, whether synthetic glycomimetics differ in functionality or may even enhance reparative process, and the optimal pathway towards clinical translation. Bhuiyan and co‐authors address these by reviewing advanced methods of synthetizing heparan sulfate glycomimetics, by summarizing the knowledge about treatment mechanisms, and by providing an outlook on potential future preclinical and clinical research programs using bioengineered extracellular matrix components. After a detailed introduction to the field, the review provides a comprehensive overview on glycosaminoglycan (GAG) chemical structure and biosynthesis, including aspects such as structural heterogeneity and resulting differences in functionality that may impact therapeutic applications. The paper then reviews the physiological roles of glycosaminoglycans in the CNS, focusing on both regeneration‐permissive and inhibitory functions. The paper further explains the interaction between glycosaminoglycans and the neurovascular unit in the context of pathophysiological events and disease, the role of the extracellular matrix and perineuronal nets in aging, and describes the functions and effects of glycosaminoglycans in neuroregeneration. The work also provides a detailed discussion of the therapeutic potential of heparan sulfate glycomimetics as well as of ongoing clinical trials. It concludes by providing an outlook about future therapeutic applications and related challenges, and by outlining future research directions. The paper also benefits from many content‐rich yet intelligible figures and tables summarizing the main content for easy reader access.

Alzheimer's disease (AD) is the leading cause of cognitive decline and the most prevalent form of dementia for which still no causative therapy is available. Thus, there is an urgent need to investigate novel approaches to augment our therapeutic arsenal against AD.^5^ Circadian rhythms are increasingly recognized as contributors to many CNS diseases^6^ including AD. Singh et al. review the current literature on that topic. It is well known that AD is characterized by disruptions of circadian rhythms and sleep. Beyond that, contemporary research also suggests that circadian dysfunction may play important roles in AD pathogenesis. For instance, clock genes were found to interact with the inflammatory process and oxidative stress and may also influence proteostasis, which is relevant for both amyloid β and tau pathologies. This may establish disruptions of circadian rhythms as a potentially modifiable pathophysiological factor in AD. The review starts with an explanation of circadian rhythms and their disruptions in the context of AD, mainly focusing on established and clinically well‐described sleep pattern alterations. It also explains the role of circadian rhythms in molecular degradation pathways. The paper then establishes mechanistic links between circadian rhythms and neurodegeneration on a molecular and cellular level. It also suggests a bi‐directional feedback loop between circadian rhythms, the suprachiasmatic nucleus, an important regulator of sleep‐wake cycles, and AD. Singh et al. further provide concepts for using early disturbance in circadian biology as potential AD biomarkers and describe potential chronotherapeutic targets. The review concludes with an outlook of potential chronotherapies for AD and related research, which must also consider systemic comorbidities being relevant for AD and their impact on circadian rhythms.

While the Severe Acute Respiratory Syndrome Coronavirus 2 (SARS‐CoV‐2) pandemic is fortunately over, clinical research now investigates post‐acute sequelae of the disease with fatigue and cognitive decline being of major interest.^7^ Qiu et al. present results from a large‐scale, multicenter, longitudinal cohort study enrolling over 3400 patients diagnosed with COVID‐19. The study specifically investigated cognitive decline after an infection with the Omicron variant of SARS‐CoV‐2. Cognitive function was assessed 6 and 24 months after infection and comprised different cognitive domains. Domain‐specific performance was analyzed, and potential risk factors for irreversible cognitive decline were identified. Cognitive dysfunction after Omicron infection comprised the domains attention/calculation, executive function, recall, registration, and learning. However, most patients showed stable cognitive functions or experienced a reversible cognitive decline. Only 3.6% of the patients exhibited progressive cognitive decline. High age, reinfection, lack of sufficient vaccination, prolonged hospitalization, lower education, hypertension, and white matter lesions were identified as risk factors of progressive cognitive decline with the later potentially being associated with cerebral small vessel disease as an independent and frequent cause of cognitive decline. Based on a very well characterized patient population, this clinical study provides highly relevant insights into the characteristics of post‐Omicron infection cognitive dysfunction, including risk factors for progressive cognitive decline.

Parkinson's disease (PD) is well known for its distinctive motor symptoms, but it also involves progressive gray matter (GM) atrophy.^8^ The latter is still incompletely understood. GM atrophy in PD can follow stage‐specific patterns which could be assessed by longitudinal imaging. Ji and colleagues conducted an imaging‐based analysis and present a study that provides detailed insight into these stage‐specific patterns of GM atrophy. The work also explores potential links to transcriptional profiles. Specifically, the study was performed in a well‐sized cohort of 542 PD patients from the Parkinson's Progression Markers Initiative. The study included patients with early‐ (<2.5 years), mid‐ (2.5–5.0 year), and late‐stage PD (>5.0 years of PD history). There were several interesting key findings. Mid‐stage PD patients showed GM atrophy in the right angular, the superior temporal, and the middle frontal gyri. In late‐stage patients, GM atrophy was further detected in the left angular, the middle occipital, and the bilateral posterior cingulate gyri as well as in the left temporal pole. Exploratory transcriptomic analyses revealed differential gene expression in hundreds of genes, many of which were differentially regulated across all three PD stages. There were also stage‐specific differential gene expressions. Gene enrichment analyses suggested specific functional changes at different PD stages. Although the presented results are mainly exploratory and descriptive, the reported findings may be valuable for informing future studies working towards the identification of mechanistic links that may not only help to better understand GM atrophy in the context of PD, but may also be used for novel prognostic and, potentially, therapeutic approaches.

Similar to stroke, traumatic brain injury (TBI) pathophysiology includes complex secondary injury cascades. Neuroinflammatory processes, including the interaction of the lesioned brain with the peripheral immune system, are a main driver of such secondary injuries, which substantially contribute to the overall functional deficit after TBI.^9^ Although neuroinflammatory processes are a potential therapeutic target with a time window being considerably longer than those of therapies targeting primary injury mechanisms, cellular and molecular responses during neuroinflammatory processes are still incompletely understood. Importantly, microglial and astrocytes can show different polarization stages that are commonly believed to exert detrimental (microglial M1 and A1 stages) as well as reparative (M2 and A2) processes. Although this dichotomous system may not fully capture the complex reality of cellular responses after TBI, it is an important first step towards a better understanding of these. Yu and co‐workers present a study that aimed to characterize responses and phenotypic shifts in microglia and astrocytes. This was not only done for acute and subacute TBI stages but also for injuries of varying severity. TBI was caused by the controlled cortical impact model, which is the established standard in the field. As expected, TBI severity was positively correlated with lesion volume and was also a good predictor for functional recovery. More severe lesions caused stronger functional deficits and only allowed for partial recovery over the observation time. There was also a clear relationship between TBI severity and the neuroinflammatory response. Mild TBI predominantly caused transient, reparative M2/A2 responses, whereas severe TBI caused an early‐onset and sustained, proinflammatory M1/A1 response. It also caused a strong increase of pro‐inflammatory cytokines in the systemic circulation. Minocycline, a potent anti‐inflammatory drug, was able to mitigate severe TBI and partially improved functional outcome. The manuscript provides valuable mechanistic insights into the pathobiology of post‐TBI neuroinflammatory responses.

Single‐cell tracking and the characterization of cell cycle stages coupled to cell migrations are meaningful readouts for imaging‐based phenotypic cellular assays. However, long‐term, high‐throughput datasets are challenging to analyze due to technical and analytical limitations. Chandra et al. report an open workflow for tracing cell cycle and motility using fluorescent ubiquitination‐based cell cycle indicator (FUCCI) reporters, which allow long‐term assessment of these parameters. Image analysis was based on well‐established freeware tools, that is, Fiji and ImageJ. The approach was shown to allow tracking of cell cycle state and migration dynamics for at least 24 h under different culture conditions. Moreover, it largely reduced false‐positive and false‐negative errors. Being applicable for single‐cell but also population‐level analyses, the approach enables the detection and quantification of several aspects related to cell migration while at the same time assessing FUCCI‐defined cell‐cycle progression and cell division. The methodological approach is described in detail, and the work is supported by comprehensive supplements, making the method easily available for other labs.

The current issue of *Neuroprotection* is concluded by a commentary written by Matute and Pacheco reviewing the scientific achievements of Nicolás Achúcarro, a Spanish clinician‐neuroscientist who passed away prematurely at the age of only 37 years after suffering from a severe hemato‐oncological disease in 1918. The main achievement of Dr. Achúcarro was to recognize the structural diversity and reactivity of neuroglia, a now outdated umbrella term summarizing non‐neuronal cell populations. He proposed that these cells play active roles in the steady state and disease and are not, as commonly believed at the time, passive support elements in the CNS. He shaped many important concepts that are still valid today, including neuroinflammatory responses and glial reactivity. Dr. Achúcarro founded a laboratory dedicated to histopathology of the CNS and developed innovative staining methods, some of which are still used nowadays. Overall, his work was truly pioneering and innovative in nature, providing the foundation of our modern understanding of glial cells. Although Dr. Achúcarro could not complete his work due to his early death, he trained an influential next generation of experimental neuropathologists who would further develop his concepts. The commentary is both interesting and valuable as it highlights the achievements of an underrecognized early architect of CNS histopathology whose work, though influential and decisive for many later generations, is often overshadowed by the achievements of other researchers of his time, such as Ramón y Cajal.

The current issue of *Neuroprotection* features a wide range of topics, which is in line with the journal's broad scope. As with previous issues, there is a good mix of review and original articles as well as on basic, translational, and clinical research. Thus, we hope that the issue will again be of much interest to our valued readership. We are especially delighted that the spectrum of articles is further diversified by a technical report, an additional editorial, and a commentary.

In the previous issue of *Neuroprotection*, we announced that the journal's first impact factor will be assigned soon.^10^ Only a few days after publishing that issue, we received our debut impact factor of 4.1. This was above expectations, and we want to thank the authors of previously published articles for their interesting and obviously impactful contributions. Editors and journal staff are now working hard to consolidate and, if possible, further increase the impact factor by strict article quality management. We also received a steeply increasing number of submissions since the debut impact factor was announced. Unfortunately, this will mean that our rejection rate is likely to increase. In order to keep submitting papers to *Neuroprotection* an attractive opportunity, we will continue the free format submission policy, meaning that first submissions can come in different formats and that meeting the journal's stylistic requirements can be achieved during any revisions. We are also happy to announce that we will further support our community by waiving article processing charges beyond the original deadline of December 2026. Publishing in *Neuroprotection* will be entirely for free until December 2027! We are confident that this will help to attract even more high‐quality submissions, which in turn will help to further build the journal's reputation and impact.

## CONFLICT OF INTEREST STATEMENT

X.J., P.W., and J.B. are Co‐Editors in Chief of *Neuroprotection*. S.L. serves as an executive editor for the journal. All authors were blinded from reviewing or making decisions on the manuscript. The article was subject to the journal's standard procedures, with peer review handled independently of the Co‐Editors in Chief and their research groups. P.W. is a founder and holds equity in IntraArt, LLC and Ti‐com, LLC.
